# Interface conditions of roughness-induced superoleophilic and superoleophobic surfaces immersed in hexadecane and ethylene glycol

**DOI:** 10.3762/bjnano.8.250

**Published:** 2017-11-27

**Authors:** Yifan Li, Yunlu Pan, Xuezeng Zhao

**Affiliations:** 1 Key Laboratory of Micro-Systems and Micro-Structures Manufacturing, Ministry of Education and School of Mechatronics Engineering, Harbin Institute of Technology, Harbin, 150001, P.R. China

**Keywords:** boundary slip, roughness, superoleophilic, superoleophobic

## Abstract

Interface conditions are an important property that can affect the drag of fluid flow. For surfaces with different oleophobicity, the boundary slip at the solid–oil interface is mostly larger than that at the solid–water interface. Roughness is a key factor for the wettability of superoleophilic/superoleophobic surfaces, and it has been found to affect the effective value of slip length in measurements. Moreover, there are no studies on the effect of roughness on slip at interfaces between oil and superoleophilic/superoleophobic surfaces. A theoretical description of the real surface roughness is yet to be found. Results show that the effective slip length is negative and decreases with an increasing root mean squared (RMS) roughness of surfaces, as the increasing roughness enhances the area with discontinuous slip at the solid–liquid interface. The underlying mechanisms are analyzed. The amplitude parameters of surface roughness could significantly inhibit the degree of boundary slip on both superoleophilic surfaces in Wenzel state and superoleophobic surfaces in Cassie state immersed in oil. The oleic systems were likely to enhance boundary slip and resulted in a corresponding reduction in drag with decreasing roughness on the solid–oil interfaces.

## Introduction

In micro/nanofluidic systems, the increasing surface to volume ratio leads to unignorable fluid drag at the solid–liquid interface. The reduction of fluid drag is an important issue to improve the efficiency of liquid delivery in confined systems in biological, chemical and medical applications [1]. Interface conditions can affect fluid drag in micro/nanofluidic systems. First introduced by Navier, the slip boundary condition in hydrodynamics suggests that the velocity of fluid flow at a solid–liquid interface is not zero. There is a relative motion that can be expressed by the so-called slip length [2]. Previous studies have shown that the presence of boundary slip leads to lower drag for the fluid flow in micro/nanochannels [3–5]. Boundary slip has been studied experimentally and theoretically on hydrophobic and oleophobic surfaces and measured slip lengths were reported by many groups [6–14].

At the micro or nano-scale, surfaces roughness can affect the liquid flow on a surface. The effect of roughness on the slip length has been investigated by theoretical and experimental studies. There are two opposing viewpoints on the effect of roughness on boundary slip. One viewpoint is that increasing roughness inhibits the degree of boundary slip, and another is the opposite. A representative summary of slip-length measurement results with varying degree of roughness is provided in Table 1. For the influence of roughness, the Léger group [15] studied slip lengths on octadecyltrichlorosilane (OTS) and incomplete self-assembled monolayers (SAM) immersed in hexadecane by using total internal reflection and fluorescence recovery after photo-bleaching (TIR-FRAP). They reported that the slip length was inhibited by roughness at the micro-scale and effective friction increased. At the nano-scale, Schmatko et al. [16] studied boundary slip on the surfaces with varying coverages of grafted nanoparticles. They demonstrated that the slip length of hexadecane decreases from 150 nm to about 0 nm as the roughness of a partially wetting surface increases at the nano-scale. Zhu and Granick [17] studied boundary slip on chemically modified octadecyltrichlorosilane (OTS) and octadecyltriethoxysilane (OTE) samples with varying degrees of roughness immersed in DI water and tetradecane by using a surface forces apparatus (SFA). They reported an inhibition of slip length with the increase of root mean squared (RMS) roughness, which suggests that a smoother surface results in a larger slip length. The Craig group [18] utilized atomic force microscopy (AFM) to measure the slip length on silicon wafer substrates roughened by treatment with KOH immersed in DI water. The result shows that slip length increases with roughness on a polymer-free surface. A similar result was reported by Guriyanova and co-workers [19]. They studied the slip length of nano-patterned silicon surfaces with varying degrees of roughness, and demonstrated that surface roughness at different length scales seems to inﬂuence the additional slippage. Kunert and Harting [20] investigated the problem of roughness-induced slip by means of lattice Boltzmann (LB) simulations and measured the slip length on a randomly generated surface with Gaussian distribute heights, and reported that slippage is independent of the detailed surface profile, and the degree of slippage enhances as the surface amplitude increases. The Bhushan group [13,21] studied boundary slip on a surface coated with SiO_2_ particle composites of varying degrees of roughness by using AFM. Results show that the slip length enhanced with larger RMS roughness, as the increasing roughness could magnify the intrinsic wetting properties of surface. In addition, the published studies based on LB simulation and molecular dynamics (MD) also show that roughness could affect slip length on wall surfaces [22–24].

**Table 1:** Summary of experimental studies on the effect of roughness on slip length.

substrate and roughness	liquid	slip-length measurement technique	slip length (nm)	trend of slip length with increasing roughness

bare sapphire (RMS ca. 0.4 nm)	hexadecane	TIR-FRAP [15]	ca. 175 nm	↓
OTS (RMS < 0.4)			ca. 150 nm	↓
OTS (RMS ca. 6 nm, ca. 3.5 nm)	DI water	SFA [17]	ca. 0 nm, ca. 5 nm	↓
OTE ( RMS ca. 2 nm, ca. 0.2 nm )			ca. 22nm, ca. 36 nm	↓
OTS (RMS ca. 6 nm, ca. 3.5 nm)	tetradecane		ca. 0 nm, ca. 6 nm	↓
OTE ( RMS ca. 2 nm, ca. 0.2 nm )			ca. 18nm, ca. 33 nm	↓
silicon wafer treated with KOH (RMS ca. 0.7 nm, ca. 4.0 nm, ca. 12.2 nm)	DI water	AFM [18]	ca. 0 nm, ca. 135 nm, ca. 900 nm	↑
grafted nanoparticles (RMS ca. 0.4 nm to ca. 30 nm)	hexadecane	TIR-FRAP [16]	ca. 150 nm to ca. 50 nm	↓
gold-coated glass (RMS ca. 4 nm to ca. 55 nm)	DI water	SFA [20]	ca.5 nm to ca.80 nm	↑
plastic disk (RMS ca. 10 nm, ca. 404 nm, ca. 770 nm)	DI water	spinning disk [25]	ca. 0 nm, ca. 580 nm, ca. 2700 nm	↑
silicon wafer (RMS ca. 0.4 nm, ca. 1.7 nm)	DI water	AFM [19]	ca. 23 nm, ca. 75 nm	↑
PS (RMS ca. 0.28 nm )	DI water	AFM [12]	ca. 0 nm	↓
OTS (RMS ca. 0.09 nm )			ca. 36 nm	↓
SiO_2_ and fluorinated acrylic copolymer (RMS ca. 57 nm, ca. 82 nm)	DI water	AFM [21]	ca. 150 nm, ca. 300 nm	↑
SiO_2_ and fluorinated acrylic copolymer (RMS ca. 57 nm, ca. 82 nm)	hexadecane		ca. 350 nm, ca. 1350 nm	↑
SiO_2_ and fluorinated acrylic copolymer (RMS ca. 57 nm, ca. 82 nm)	ethylene glycol		ca. 600 nm, ca. 1800 nm	↑

Although roughness is believed to have effects on boundary slip, a complete theoretical description of the relationship between realistic surface roughness and slip is yet to be found. Previous studies focused on the interface conditions of hydrophilic, hydrophobic, oleophilic and oleophobic surfaces, and there is no data for superoleophilic and superoleophobic surfaces. In superoleophobicity, the values of the roughness parameters are mostly larger than those of surfaces with hydrophobicity/oleophobicity. As oils are widely used as lubricants and antifreeze in micro/nanofluidic systems, the effects of roughness on the boundary slip at the solid–oil interface are of scientific interest. However, the relationship between roughness at a large scale and interface conditions is yet to be found. As described in [26], there are several issues of experimental studies that need to be addressed. Firstly, fabricating surfaces with controlled roughness parameters, omniphobicity and interface properties is difficult. Roughness parameters, wettability and some other surface properties have individual effects on the boundary slip and need to be separated. Secondly, the scale effect of roughness influences the values of boundary slip. Surfaces at different scales need to be discussed individually. Finally, the position of the boundary needs to be determined firstly when investigating boundary slip, after that the position of the reference surface needs to be defined. When measuring the slip length by using AFM and SFA, the set of reference surfaces can lead to a shift of the boundary and result in different calculated values of the effective slip length, as pointed out by the Vinogradova group [27] and the Craig group [28]. If the reference surface is located on the peaks of the rough surface, the calculated value of the measured slip length will tend to be larger than the true value as the liquid molecules can still flow between the peaks and valleys. Thus, studies on the boundary slip of surfaces with various degrees of roughness must locate the position of the reference surface. For techniques in which hydrodynamic forces are used to derive the slip length, the measured slip length should be replaced by the effective slip length.

In this paper, the interface conditions of superoleophilic and superoleophobic surfaces at the solid–oil interface were studied. The effects of surface roughness on the boundary slip of surfaces immersed in oils were analyzed, and the mechanisms were discussed. Series of multilayered composite surfaces with variable roughness parameters and identical chemical properties were fabricated, and then were used to investigate the effect of roughness on the slip length. To decouple the individual effects of roughness and wettability on slip, the contact angles were kept constant. The roughness parameters were measured by using a laser confocal scanning microscope, and the boundary slip measurements were carried out using an AFM with a colloidal probe in contact mode. Reference surface of superoleophilic and superoleophobic samples were defined and the effective slip lengths were obtained. The situation of superoleophilic surfaces in Wenzel state and superoleophobic surfaces in Cassie state were discussed in this paper.

## Experimental

At first, the preparation for surfaces with controlled roughness parameters for the boundary-slip studies is introduced. Then the measurement techniques of surface roughness, wettability and slip length are described. Finally, the location of reference surface is discussed and the modification for the calculation of effective slip length is presented.

### Preparation of surfaces and liquids

**Figure 1:**
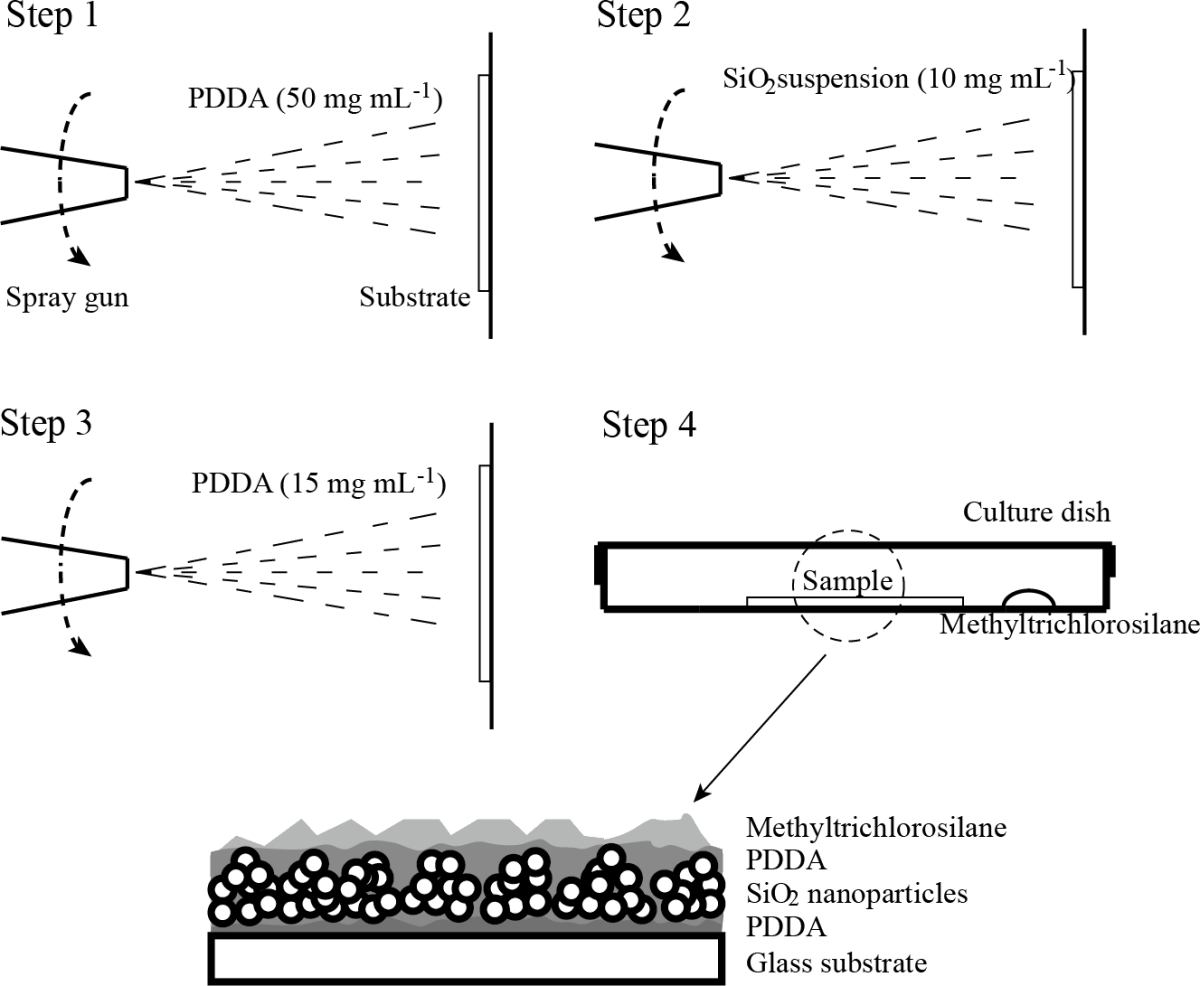
Schematic illustration of the preparation procedures for the superoleophilic samples. The dashed arrow is the moving direction of the spray gun, which is parallel to the samples.

For the preparation of the superoleophilic surface, multilayered composite surfaces were synthesized [29]. Soda-lime glass (CAT. NO. 7101, SAIL BRAND, China) with 1.0 mm thickness was used as substrates. Poly(diallyldimethylammonium chloride) (PDDA, *M*_W_ = 100,000–200,000, Aladdin) was dissolved in DI water to concentrations of 15 and 50 mg·mL^−1^. SiO_2_ nanoparticles with a diameter of 7 nm (AEROSIL RX 50, Evonik Industries) were dispersed into acetone (Fisher Scientific) at various concentrations (10 to 30 mg·mL^−1^). The suspension was sonicated with a Sonifier homogenizer (JY96-IIN, Scientz Company, China) at a frequency of 20 kHz, an amplitude of 30% and a duty circle of 50% (5 s). After the preparation of the solutions, a spray gun (Airbrush-S130, U-star Company, China) was used to deposit the solutions on the glass substrate. The spray gun was held vertically at a distance of 15 cm from the glass substrate and a pressure of 200 kPa was applied (Figure 1). For the multilayered composite coating, three steps of spray depositions were applied. First, PDDA solution (50 mg·mL^−1^, 2 mL) was spray coated (thickness ca. 200 nm). Second, the SiO_2_ suspension (10 to 30 mg·mL^−1^, 2 mL) was spray coated (thickness ca. 200 to 350 nm). Third, a second PDDA layer was deposited (15 mg·mL^−1^, 2 mL, thickness ca. 50 nm). After this, the samples were annealed in an oven at 140 °C for 1 h. Finally, to form the function layer (thickness ca. 20 nm) by chemical vapor deposition (CVD), one drop (about 0.1 mL) of methyltrichlorosilane (methylsilane, Sigma Aldrich) was placed next to the samples under sealing and left for 10 h.

To prepare the superoleophobic surface, the same materials and processes as for superoleophilic samples were used, except for the deposition of the functional layer. Fluorosurfactant solution (FL, Capstone FS-50, DuPont) was diluted with ethanol (Decon Labs) to a concentration of 45 mg·mL^−1^, then the solution was spray-coated to form a functional layer (thickness ca. 30 nm). The samples were dried in air and left for 10 h.

There were ten groups of samples: “a1” to “a5” with superoleophilic surfaces and “b1” to “b5” with superoleophobic surfaces. The concentration of the SiO_2_ suspension increased from a1 to a5 and b1 to b5, respectively.

The properties of the liquids used in the experiment are shown in Table 2. Hexadecane and ethylene glycol were selected to study the interface conditions at the solid–oil interface on the superoleophilic and superoleophobic surfaces.

**Table 2:** Properties of the liquids used in the experiments [30].

liquid	density (g/cm^3^)	surface tension (mN/m)	dynamic viscosity (mPa·s)

hexadecane	0.7701	27.05	3.032
ethylene glycol	1.1135	47.7	16.100

### Characterization of the surfaces

#### Roughness of the samples

The morphology of the superoleophilic and superoleophobic surfaces in air was measured by using a laser confocal scanning microscope (OLS 3000, Olympus, Japan) at a magnification of 100 in 3D scanning mode. The surfaces were imaged with scan size of 128 μm × 128 μm at a step length of 10 nm. The surface roughness can be obtained from the OLS 3000 measurement.

**Figure 2:**
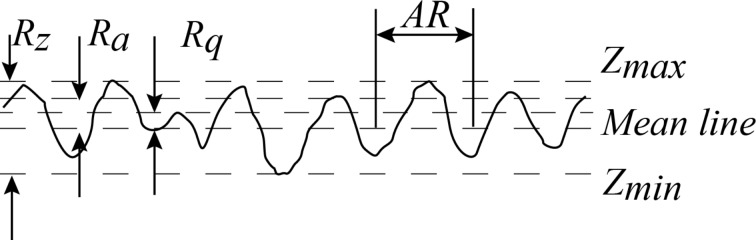
Schematic illustration of a rough surface. The parameters of the profile defined here are the mean line, the average width of the roughness pitch (*AR*), the peak to valley roughness (*R_z_*), the average roughness (*R_a_*), the root mean squared roughness (*R_q_*), the height of peaks (*Z_max_*) and height of valleys (*Z_min_*).

Surface roughness is most commonly referred to the width and height of the surface relative to a reference plane [31]. It can be characterized by the pitch parameters and amplitude parameters. A schematic illustration of a rough surface is shown in Figure 2. The most commonly used pitch parameters are average width of the roughness pitch (*AR*) and the average wavelength (*AW*) of roughness motifs. The amplitude parameters include the maximum depth (*R_z_*), the arithmetical mean deviation (*R_a_*), and the RMS deviation (*R_q_*) of profile irregularity. *R_q_* roughness, which is defined as the RMS value of the ordinate values within a sampling length, is selected to describe the amplitude of roughness in this paper as it is more sensitive than *R_a_* to large deviations from the mean line:

[1]
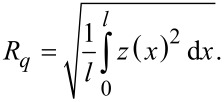


The mean line is determined by equating the area enclosed by the profile of the surface above and below the line [26].

An increasing concentration of silica nanoparticles in the composite surfaces from 10 to 30 mg·mL^−1^ leads to an increase in the amplitude parameters, while the pitch parameters remain random. To decouple the individual effect of the amplitude parameters and pitch parameters on slip, surfaces with the same *AR* roughness were selected.

#### Contact angle and contact angle hysteresis measurements

Surface wettability can affect boundary slip. In order to decouple the individual effects of roughness and wettability on slip, the contact angle (CA) and contact angle hysteresis (CAH) have to be constant. CA and CAH were measured by using a goniometer (DropMeter^TM^ Element A-60, MAIST Vision Inc., China) and the Dropmeter software. For CA measurements, a droplet with a volume of 5 μL of each liquid was deposited on the prepared surface. The measurements were taken with three droplets at different positions for one surface, and the goniometer collects six values for each droplet, from which the average CA is obtained. For CAH measurements, a droplet of 5 μL of each liquid was deposited on the sample and was captured by the goniometer. Then, by using a syringe a volume of 1 μL was added until swelling deformation. The swelling droplet was captured by goniometer to obtain the CA. The difference between the CAs of the droplets was calculated as the CAH. On two more positions at the surface, the operation was repeated two times to obtain the average CAH.

### Boundary-slip measurements using an AFM

#### Boundary slip measurements

**Figure 3:**
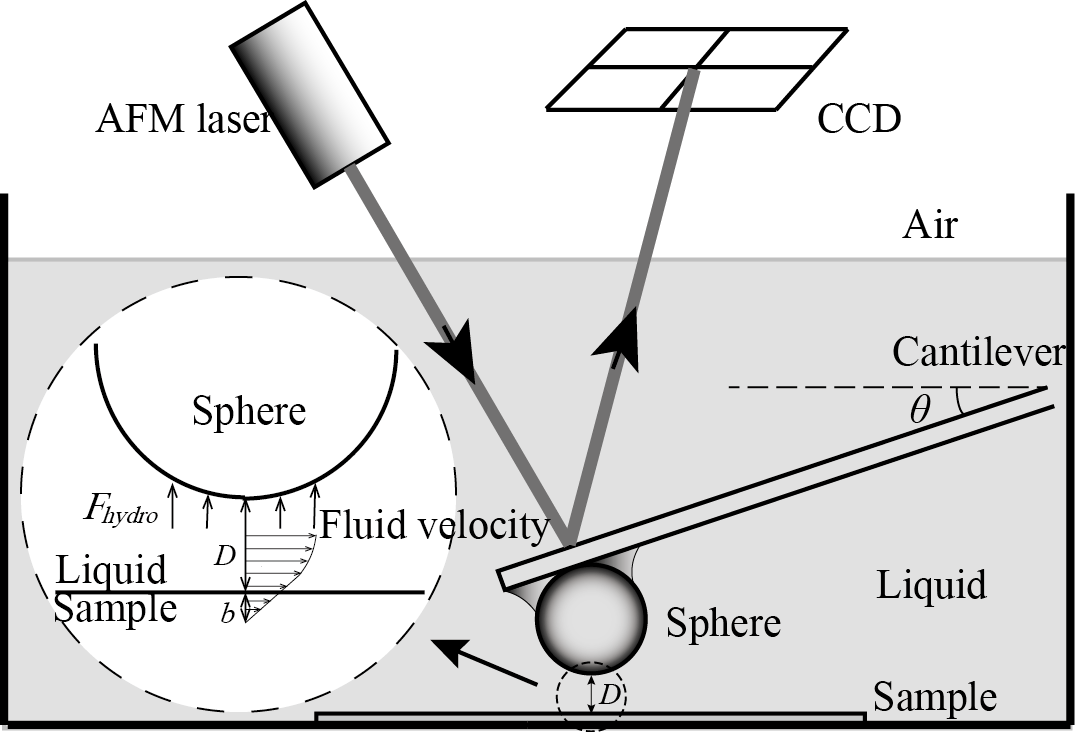
Schematic illustration of a slip-length measurement by using a colloidal AFM probe in contact mode. With the approach of the sphere towards the sample, the liquid is set into fluid flow. The arrows above and below the solid–liquid interface represent magnitude and direction for fluid flow with boundary slip. The definition of slip length *b* characterizes the degree of boundary slip at the solid–liquid interface, and can be obtained by the hydrodynamic force on the cantilever detected with CCD.

A colloidal AFM technique was used for the measurement of boundary slip, as shown in Figure 3. A borosilicate sphere (GL018B/45-33, MO-Sci Corporation) with a measured diameter of about 56.5 μm was glued to the end of a rectangular cantilever of an AFM tip (ORC8, Bruker) by using epoxy resin to prepare the colloidal AFM tip. Then the probe was driven towards the surface immersed in liquid at a certain driving velocity. By analyzing the force exerted on the probe, which mainly includes hydrodynamic forces, electrostatic forces, van der Waals force and Stokes force, the boundary slip can be calculated [12,18–19,21].

#### Effective boundary slip

**Figure 4:**
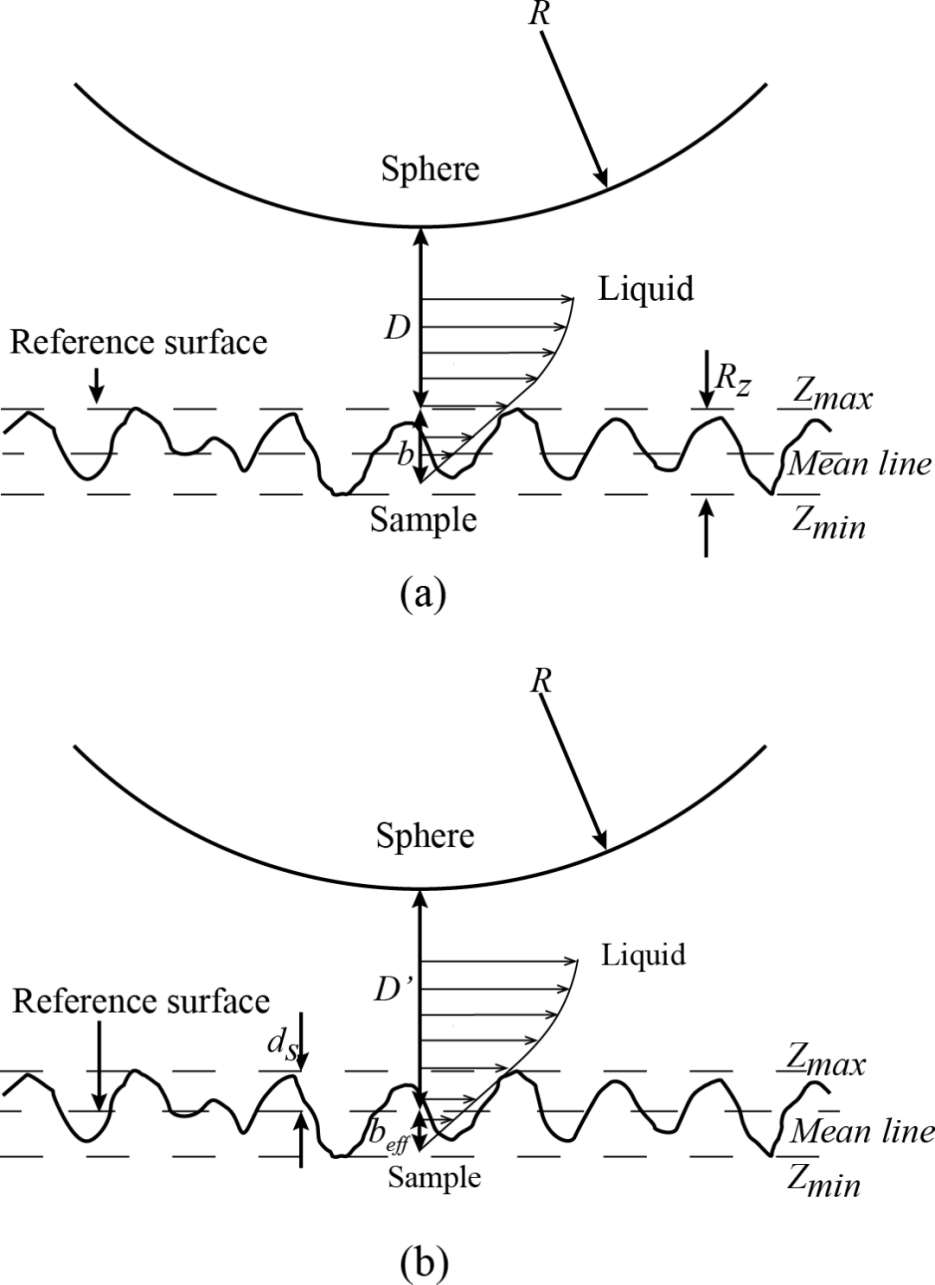
Schematic illustrating possible definitions of the boundary-slip condition at a rough surface. (a) The boundary-slip condition with the reference surface set at the peak of the sample. (b) The boundary-slip condition with the position of the reference surface set between the peaks and valleys of the sample, where *b_eff_* is the effective slip length.

For the boundary-slip study, the position of the reference surface where hydrodynamic force was used to obtain the effective slip length had to be addressed. As shown in Figure 4a, the bottom of the sphere could be treated equivalent to a flat wall as its degree of roughness is much less than that of the rough surface. When the sphere is driven to approach the surface and makes a hard contact on the top of peaks, the separation distance *D* is zero. If the reference surface is defined at the top of the peaks according to the hard contact, the results of the measured slip length would tend to be larger as the hydrodynamic force curve would be fitted at a larger separation distance. This would need to be corrected. As shown in Figure 4b, for the boundary slip on the rough surface, the boundary-slip length was corrected by shifting the reference surface by a distance *d_s_* from the peaks. It is assumed that the effective boundary, which is obtained at the position of reference surface, is at the height of *R_z_* − *d_s_*. The corrected slip-length model is required to reliably estimate the slip.

For a sphere which is close to the surface, the hydrodynamic force *V*/*F_hydro_* between a sphere and a surface can be written as

[2]
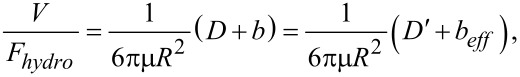


where *D*′ is the shifted separation distance and *b_eff_* is the effective slip length. In this case, the correction for boundary slip takes the form:

[3]
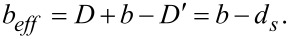


When the sphere moves approaching the surface, a Newtonian fluid flow is assumed to be between sphere and surface. Guriyanova et al. [19] set the reference surface at the bottom of the valleys and shifted the curve of the hydrodynamic force with the height of the peak to valley (P–V) roughness in the drainage experiments. Kunert and Harting [20] point out that the position of the reference surface can be found by fitting the parabolic flow profile at a height of 1.69*R_a_*. According to the study of Pan et al. [11] on the correction of slip length, the mean line of the profile is a typical case to describe surface roughness based on the ISO standard. A position within the surface near the height of the mean line on the surface would be a more reasonable choice than at the top of the peaks. Thus, the reference surface is defined as the position of the mean line. The height of asperities, *d_s_*, is defined by *b_eff_* = *b* − *d_s_*, via *d_s_* = (1/2)*R_z_* = (1/2)(*Z_max_* − *Z_min_*).

## Results and Discussion

### Morphology and surface roughness

**Figure 5:**
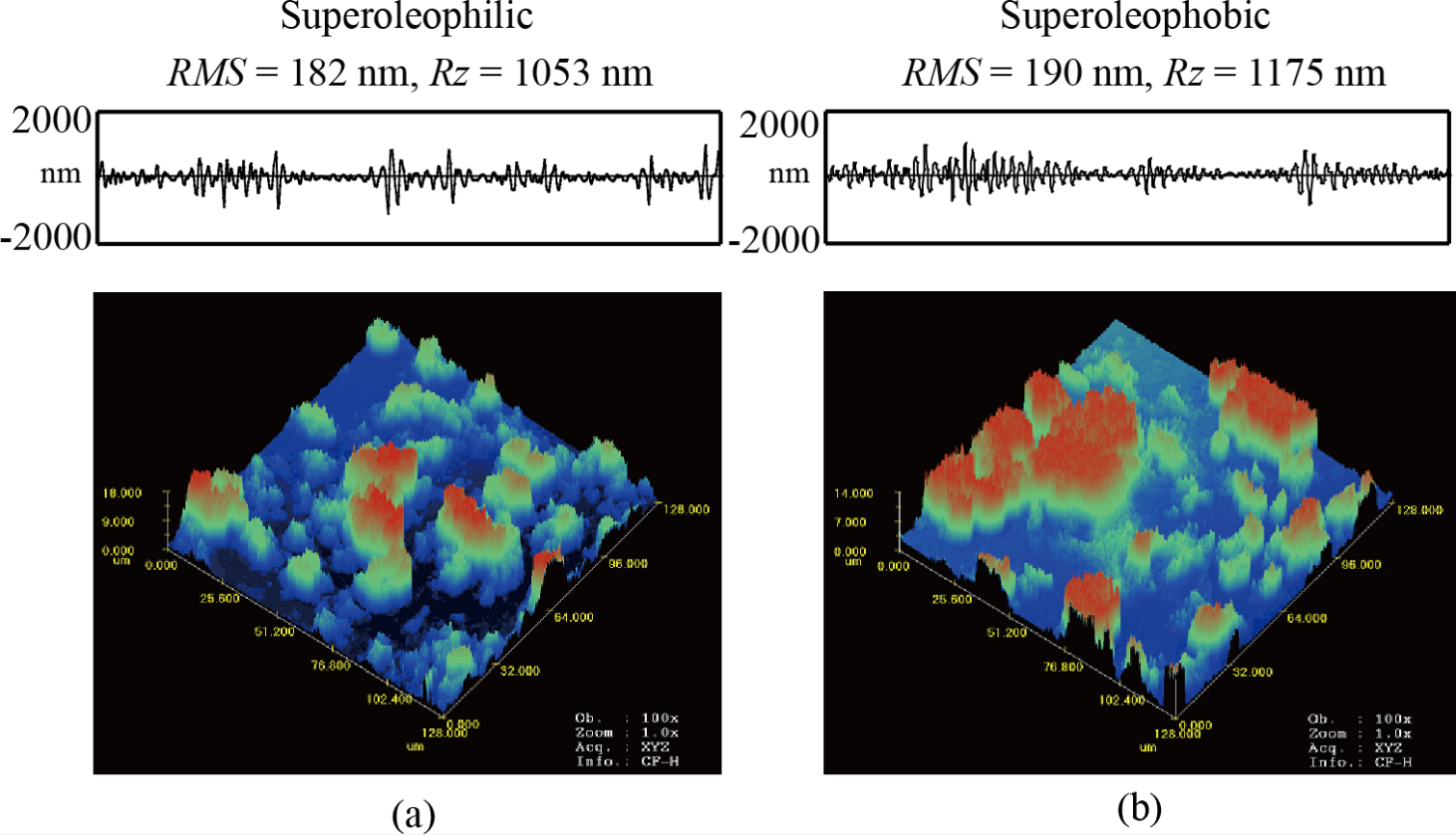
Confocal laser scanning images in the air, and measured values of RMS and *R_z_* roughness of (a) superoleophilic sample a1 and (b) superoleophobic sample b1 at 128 μm × 128 μm scan size.

Figure 5 shows the laser confocal scanning microscopy images of samples a1 and b1 in air with RMS roughness and peak to valley (P–V) distance calculated. A 128 μm × 128 μm scan size was used to present the data. For the superoleophilic and superoleophobic surface, the SiO_2_ nanoparticles coalesce together to form a random rough structure. The values of RMS and *R_z_* roughness of b1 are larger than that of a1. From the data shown below in Table 3, it can be noted that the values of RMS and *R_z_* roughness increase from a1 to a5 and b1 to b5, respectively. With increasing concentration the SiO_2_ nanoparticles coalesce to larger agglomerates and enhance the amplitude parameters of the surfaces. The average width of the roughness pitch (*AR*) is kept constant at 2000 ± 100 nm.

### Contact angle and contact angle hysteresis

Table 3 shows the CAs and CAHs of hexadecane and ethylene glycol on the superoleophilic and superoleophobic surfaces. For the superoleophilic surfaces, CAs and CAHs of the hexadecane droplet remain at 0° and are smaller than those of ethylene glycol of ca. 10° with increasing RMS roughness from a1 to a5.

**Table 3:** *R_z_* and RMS roughness, and CA and CAH of the superoleophilic and superoleophobic surfaces.

sample	*R_z_* roughness (nm)	RMS roughness (nm)	hexadecane				ethylene glycol			
			CA (°)	θ_A_^a^ (°)	θ_R_^a^ (°)	CAH (°)	CA (°)	θ_A_^a^ (°)	θ_R_^a^ (°)	CAH (°)

superoleophilic										

a1	1090 ± 200	180 ± 20	0	—	—	—	10 ± 1	—	—	—
a2	1400 ± 180	204 ± 30	0	—	—	—	10 ± 1	—	—	—
a3	1850 ± 350	280 ± 30	0	—	—	—	10 ± 1	—	—	—
a4	2200 ± 450	305 ± 40	0	—	—	—	10 ± 1	—	—	—
a5	2700 ± 300	323 ± 30	0	—	—	—	10 ± 1	—	—	—

superoleophobic										

b1	1200 ± 300	190 ± 40	150 ± 3	153 ± 2	144 ± 3	10 ± 2	159 ± 5	161 ± 1	154 ± 2	7 ± 1
b2	1800 ± 300	270 ± 50	150 ± 3	152 ± 2	145 ± 1	8 ± 1	160 ± 3	161 ± 1	155 ± 1	5 ± 1
b3	1900 ± 300	279 ± 40	152 ± 2	156 ± 2	148 ± 3	6 ± 1	158 ± 3	160 ± 1	155 ± 1	6 ± 1
b4	2100 ± 250	285 ± 50	151 ± 2	154 ± 2	145 ± 1	8 ± 1	160 ± 3	162 ± 1	157 ± 2	5 ± 2
b5	2500 ± 500	344 ± 50	155 ± 3	158 ± 1	150 ± 2	8 ± 2	158 ± 5	160 ± 3	155 ± 1	5 ± 1

^a^θ_A_ is the advancing contact angle, while θ_B_ is the receding contact angle.

For the superoleophobic surfaces, the CAs and CAHs of the hexadecane droplet on the surfaces remain at ca. 150° and are smaller than those of ethylene glycol of ca. 160° with increasing RMS roughness from b1 to b5. For each type of samples, CAs and CAHs are constant with increasing roughness and the effect of wettability is decoupled.

### Boundary slip

#### Superoleophilic surfaces

**Figure 6:**
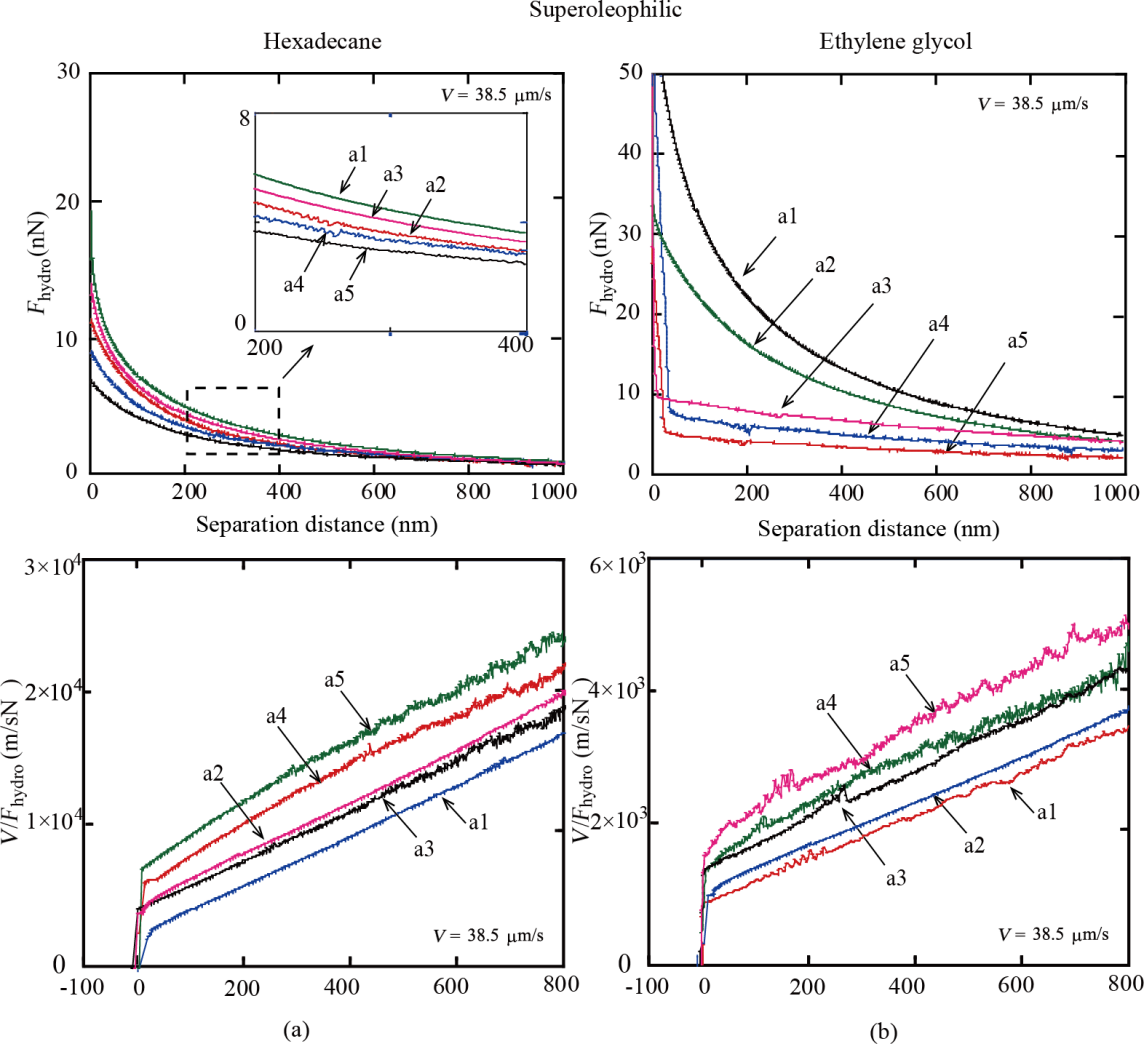
Hydrodynamic force *F_hydro_* and *V*/*F_hydro_* of a borosilicate sphere on superoleophilic surfaces a1 to a5 immersed in hexadecane with various values of RMS roughness at a sphere velocity of 38.5 μm/s.

**Hydrodynamic forces:** The plots of measured hydrodynamic forces *F_hydro_* between the colloidal AFM probe and the superoleophilic surfaces immersed in hexadecane and ethylene glycol as a function of the separation distance are shown in Figure 6. In order to obtain the slip length, the values of *V*/*F_hydro_* are presented as a function of the separation distance (Figure 6a). For hexadecane, the plots of *V*/*F_hydro_* exhibit linear behavior and shift to the left with increasing RMS roughness from sample a1 to a5 at separation distances of 0 to 800 nm. This means that the intercepts of *V*/*F_hydro_* on the separation-distance axis shift to the left from sample a1 to a5, indicating an increase of slip length. For ethylene glycol, the plots of *V*/*F_hydro_* are similar to those of hexadecane and also exhibit linear behavior (Figure 6b). It can be noted that the slopes are smaller than those of hexadecane. This means that the slip length of ethylene glycol is larger than that of hexadecane as the results are related to the viscosity of the liquids given in Table 2.

**Figure 7:**
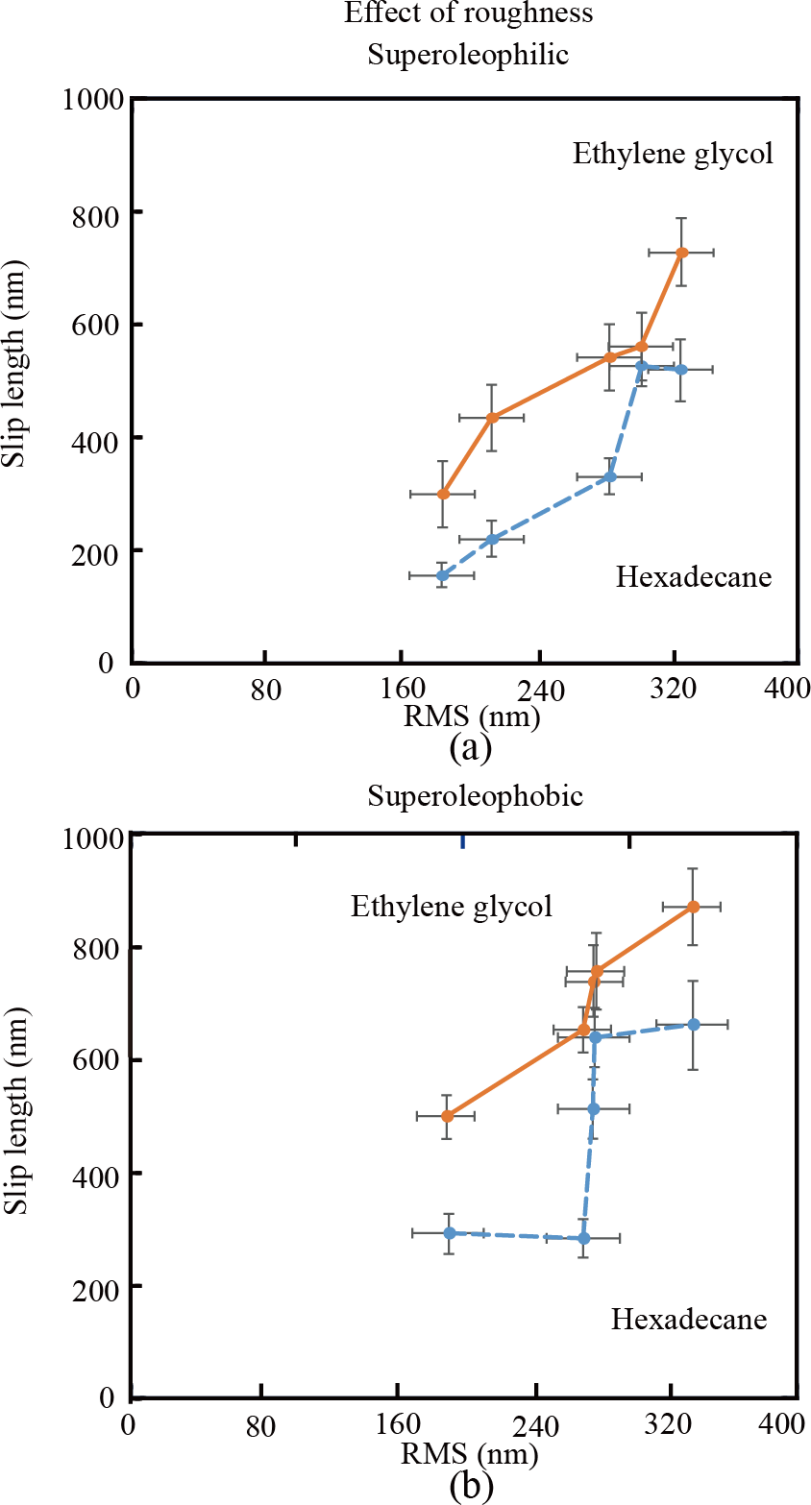
Measured slip length on (a) superoleophilic and (b) superoleophobic surfaces with varying values of RMS roughness immersed in hexadecane and ethylene glycol.

**Figure 8:**
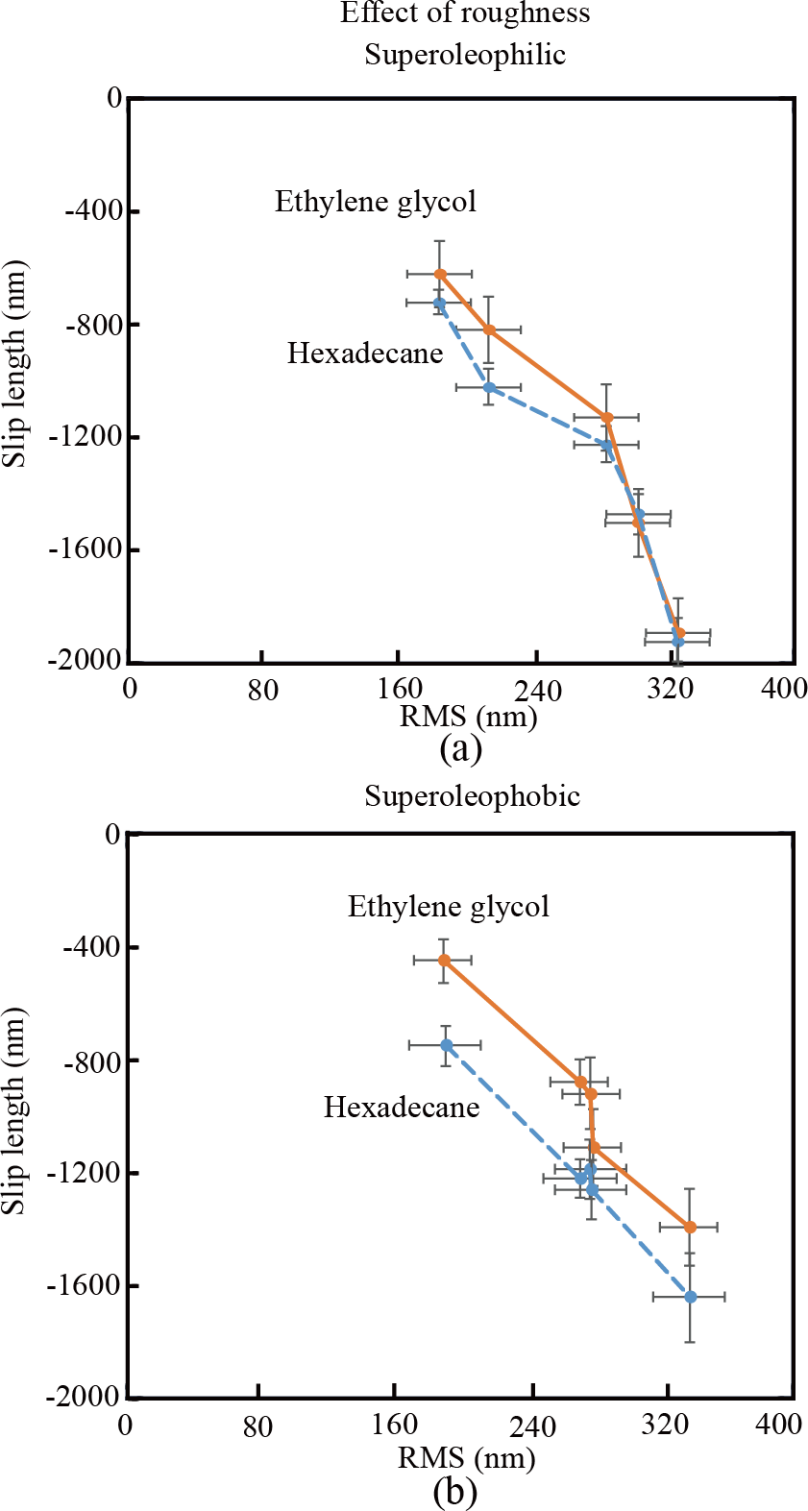
Effective slip length on superoleophilic and superoleophobic surfaces with varying values of RMS roughness immersed in hexadecane and ethylene glycol.

**Slip length:** The slip length is obtained from the hydrophobic forces according to Equation 1. Figure 7a shows the measured slip length as a function of the RMS roughness. With the increase of roughness, the slip length of the superoleophilic surface in hexadecane and ethylene glycol increases. The effective slip length is obtained according to Equation 3. Figure 8a shows the effective slip length as a function of the RMS roughness. Results show that all slip lengths are negative and decrease from a1 to a5. This means that the flow velocity at the reference surface is discontinuous due to the rough structure. The increasing amplitude parameter enhances the fluid shear and inhibits the flow velocity, which forms an area where the average velocity equals that under non-slip conditions. As the slip length *b* is obtained through the average hydrodynamic force *F_hydro_* and its absolute value equals the height of the area, the effect of surface roughness on the slip length can be explained with the increasing roughness, which enhances the height of discontinuous area next to the reference surface. The absolute value of slip length *b* increases with increasing height, and the slip length *b* decreases, because *b* it is negative.

One can qualitatively understand that slip length on the rough superoleophilic surface is discontinuous and should be studied by the average hydrodynamic forces detected by AFM.

#### Superoleophobic surfaces

**Figure 9:**
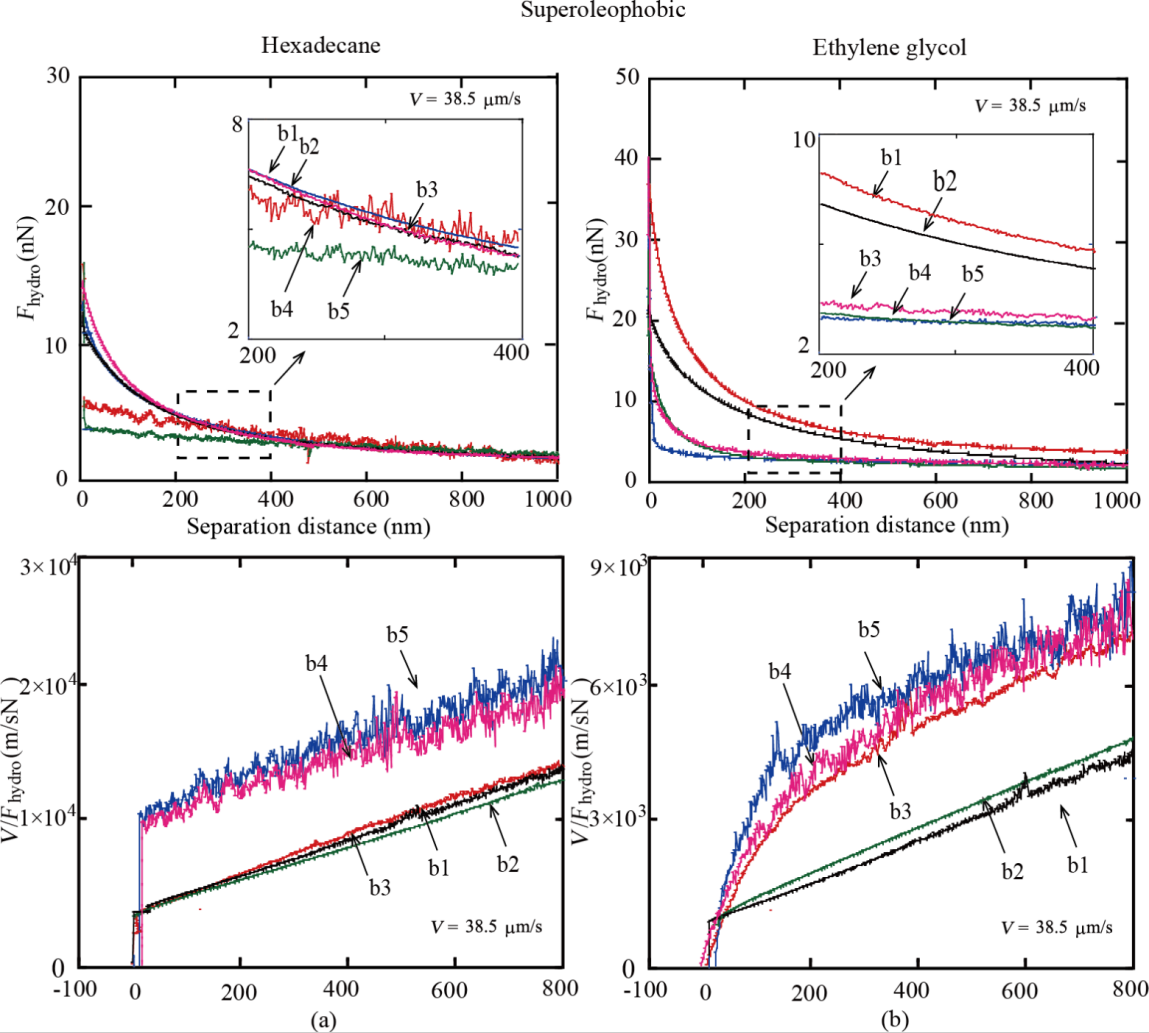
Hydrodynamic force *F_hydro_* and *V*/*F_hydro_* of a borosilicate sphere on superoleophobic surfaces b1 to b5 immersed in ethylene glycol with varying values of RMS roughness at a sphere velocity of 38.5 μm/s.

**Hydrodynamic forces:** The measured hydrodynamic forces *F_hydro_* between the colloidal AFM probe and the superoleophobic surfaces immersed in hexadecane and ethylene glycol as a function of the separation distance are shown in Figure 9. Results show that the changes with increasing RMS roughness from sample b1 to b5 are larger than those of sample a1 to a5 (Figure 6). This is related to the CAs of the superoleophilic and superoleophobic surfaces. In order to obtain the slip length, the values of *V*/*F_hydro_* are presented as a function of the separation distance. It can be noted that the fluctuation of the measured values increases from b1 to b5. This means that the error of the measurement increases with increasing roughness. For hexadecane, the plots of *V*/*F_hydro_* on superoleophobic surfaces exhibit linear behavior and shift to the left with increasing RMS roughness at separation distances of 0 to 800 nm (Figure 9a). This means that the intercepts of *V*/*F_hydro_* on the separation distance axis shift to the left from sample b1 to b5, indicating an increase of slip length. For ethylene glycol, the plots of *V*/*F_hydro_* exhibit a nonlinear behavior at separation distances of 0 to 200 nm and are almost linear at 200 to 800 nm (Figure 9b). This is related to the high viscosity of ethylene glycol. The results are similar to those of hexadecane and are indicative of an increase of slip length from sample b1 to b5.

**Slip length:** The slip length is obtained from the hydrophobic forces according to Equation 1. Figure 7b shows the measured slip length as a function of the RMS roughness. With the increase of roughness from b1 to b5, the slip length of the superoleophobic surface in hexadecane and ethylene glycol increases. The reference surface of the rough sample in a Cassie state remains an open question. It can be different in different applications. When the hydrodynamic force on the surface immersed in liquids is calculated, the reference surface should be localized at the contact line of the liquid and the air between liquid and solid. When we calculate the liquid flow in a channel with rough walls on both sides, the reference surface, which is used for calculating the height of the channel, should be located at the mean surface line as well. In this paper, the application in microfluidic channels with rough walls on both sides has been considered. Hence, so the reference surface should still be considered as the mean line of the roughness profile, which remains the same when measuring the height of the microfluidic channel. Then the effective slip length is obtained according to Equation 3. Figure 8b shows the effective slip length as a function of the RMS roughness. Results are similar to those of the superoleophilic surface. All slip lengths are negative and decrease from b1 to b5. It also can be explained by the rough topography leading to a discontinuity of boundary slip.

## Conclusion

The effect of surface RMS roughness on the interface conditions was studied by using laser confocal scanning microscopy and AFM. Experiments on superoleophilic and superoleophobic surfaces with varying values of surface RMS roughness immersed in hexadecane and ethylene glycol were carried out. Negative slip lengths were found on the superoleophilic and superoleophobic surfaces, as the viscosity of the liquids at the reference surface were discontinuous. The negative slip length decreased with increasing RMS roughness as the increasing roughness enhanced the absolute value of the effective slip length.

In conclusion, it was shown that the amplitude parameters of surface roughness could significantly inhibit the degree of boundary slip on the superoleophilic and superoleophobic surfaces immersed in oil. For fluidic systems in which liquids and surface roughness were similar to that used in this study, it is suggested that boundary slip is likely enhanced and results in a corresponding reduction of the drag with decreasing roughness.
